# Zinc antagonizes iron-regulation of tyrosine hydroxylase activity and dopamine production in *Drosophila melanogaster*

**DOI:** 10.1186/s12915-021-01168-0

**Published:** 2021-11-03

**Authors:** Guiran Xiao, Mengran Zhao, Zhihua Liu, Fan Du, Bing Zhou

**Affiliations:** 1 State Key Laboratory of Membrane Biology, School of Life Sciences, Tsinghua University, Beijing, 100084 China; 2 School of Food and Biological Engineering, Hefei University of Technology, Hefei, 230009 Anhui China

**Keywords:** *Catecholamines up*, Zinc, Iron, Tyrosine hydroxylase, Parkinson’s disease

## Abstract

**Background:**

Dopamine (DA) is a neurotransmitter that plays roles in movement, cognition, attention, and reward responses, and deficient DA signaling is associated with the progression of a number of neurological diseases, such as Parkinson’s disease. Due to its critical functions, DA expression levels in the brain are tightly controlled, with one important and rate-limiting step in its biosynthetic pathway being catalyzed by tyrosine hydroxylase (TH), an enzyme that uses iron ion (Fe^2+^) as a cofactor. A role for metal ions has additionally been associated with the etiology of Parkinson’s disease. However, the way dopamine synthesis is regulated in vivo or whether regulation of metal ion levels is a component of DA synthesis is not fully understood. Here, we analyze the role of Catsup, the *Drosophila* ortholog of the mammalian zinc transporter SLC39A7 (ZIP7), in regulating dopamine levels.

**Results:**

We found that Catsup is a functional zinc transporter that regulates intracellular zinc distribution between the ER/Golgi and the cytosol. Loss-of-function of Catsup leads to increased DA levels, and we showed that the increased dopamine production is due to a reduction in zinc levels in the cytosol. Zinc ion (Zn^2+^) negatively regulates dopamine synthesis through direct inhibition of TH activity, by antagonizing Fe^2+^ binding to TH, thus rendering the enzyme ineffective or non-functional.

**Conclusions:**

Our findings uncovered a previously unknown mechanism underlying the control of cellular dopamine expression, with normal levels of dopamine synthesis being maintained through a balance between Fe^2+^ and Zn^2+^ ions. The findings also provide support for metal modulation as a possible therapeutic strategy in the treatment of Parkinson’s disease and other dopamine-related diseases.

**Supplementary Information:**

The online version contains supplementary material available at 10.1186/s12915-021-01168-0.

## Background

Parkinson’s disease (PD) is one of the most common neurodegenerative diseases with clinical manifestation of motor dysfunctions in the form of tremor, rigidity, and bradykinesia. A hallmark of PD is the progressive dopaminergic neuron death mainly in the ventral midbrain substantia nigra pars compacta (SNc) [1]. The exact molecular cause underlying neural cell death in PD has not been fully elucidated*,* and no cure is available. It has long been believed that dopamine (DA) deficit, resulted from dopaminergic neuron death, is a key culprit of PD. DA is the key signaling neurotransmitter molecule critical to body movement, cognition, attention, and reward response [2]. Besides PD, abnormal dopaminergic signaling has also been implicated in a number of other diseases, including attention deficit hyperactivity disorder, psychosis, schizophrenia, and depression [3–5]. The entire pathway of DA synthesis has been well established, and the initial and rate-limiting step is catalyzed by the tyrosine hydroxylase (TH) [6], a metal-dependent enzyme that uses Fe^2+^ as the cofactor. TH activity is known to be regulated by a complex mechanism including phosphorylation [7]. Since alteration in TH activity and DA synthesis/signaling has been found to be associated with PD and other neurological disorders [8], modulation of TH activity or DA signaling has been proposed as a potential therapeutic strategy for the treatment of PD [9, 10].

There are ample evidences indicative of a role for metal ions in PD etiology [11, 12], consistent with the fact that the quintessential TH in DA synthesis is a Fe^2+^-dependent enzyme. Clinic manifestation of manganism, a disorder of manganese toxicity, resembles idiopathic PD as well as other neurodegenerative diseases such as Lou Gehrig’s disease and multiple sclerosis [13]. In the dopaminergic neurons of PD patients, the iron level was found to be increased in the substantia nigra but decreased in the globus pallidus [14, 15], and zinc in contrast was abnormally accumulated in the region [15]. Feeding rats with excess zinc was found to trigger degeneration of dopaminergic neurons and decreased DA levels [16, 17], implying abnormally high zinc levels in the dopaminergic neurons of PD patients might be causative. Interestingly, iron content in PD patients’ hair was significantly lowered, and zinc content in contrast was increased [18], suggesting a possible alteration and abnormal interplay between these two metal ions in these patients. Whether or not these metal-related defects are part of the underlying cause or aftermath consequences of PD remains to be more affirmatively determined. Furthermore, how these different metal ions might contribute to PD lacks a unified mechanistic understanding.

We have long been interested in understanding how metal ions might contribute to and influence PD and other neurodegenerative diseases. We were particularly interested in a *Drosophila* mutant named *Catecholamines up* (*Catsup*). As the name indicates, in *Catsup* mutant flies, the DA level was found to be substantially elevated [19]. The corresponding protein Catsup encoded by *Catsup/*CG10449 has been characterized as a negative regulator of TH activity during catecholamine biosynthesis [19]. Loss of Catsup function was shown to delay the onset of neurological symptoms, dopaminergic neuron death, and morbidity of flies upon paraquat exposure [20]. Catsup has also been found to participate in the synaptic vesicle loading and release of DA [21], the regulation of tracheal morphogenesis [22], the control of sleep behavior in *Drosophila* [23], and the maintenance of ER/Golgi function [24]. Interestingly, Catsup is homologous to the mammalian zinc transporter ZIP7 protein (also known as SLC39A7) [25], a member of the ZIP (Zrt/Irt-like protein) family [26]. Mammalian ZIP7 is also known to be localized in the ER and functions in mobilizing zinc from ER into the cytosol [26–29]. It is therefore possible that the *Drosophila* Catsup might function as a zinc transporter somehow exerting regulatory control of DA synthesis, and this likely scenario could explain how zinc may contribute to dopaminergic neural degeneration.

In the current study, we report both genetic and biochemical studies demonstrating that Catsup is indeed a functional zinc transporter participating in zinc homeostasis in *D. melanogaster*. *Catsup* knockdown leads to zinc reduction in the cytosol. More importantly, we demonstrated that cytosolic zinc reduction triggered TH activation and DA increase. We further showed that zinc affects TH activity thus DA synthesis by antagonizing Fe^2+^, the indispensable cofactor of TH. Our study has uncovered a previously unknown mechanism underlying the regulation of DA synthesis. The finding provides a mechanistic framework to understand how zinc and iron ions could affect DA synthesis therefore contribute to PD. Our finding also points to the utility of zinc limitation as a possible novel therapeutic strategy in the treatment of PD and implies a role of metal homeostasis in the regulation of mood or behavior of animals.

## Results

### Catsup is a zinc transporter functionally analogous to human ZIP7

**Fig. 1:**
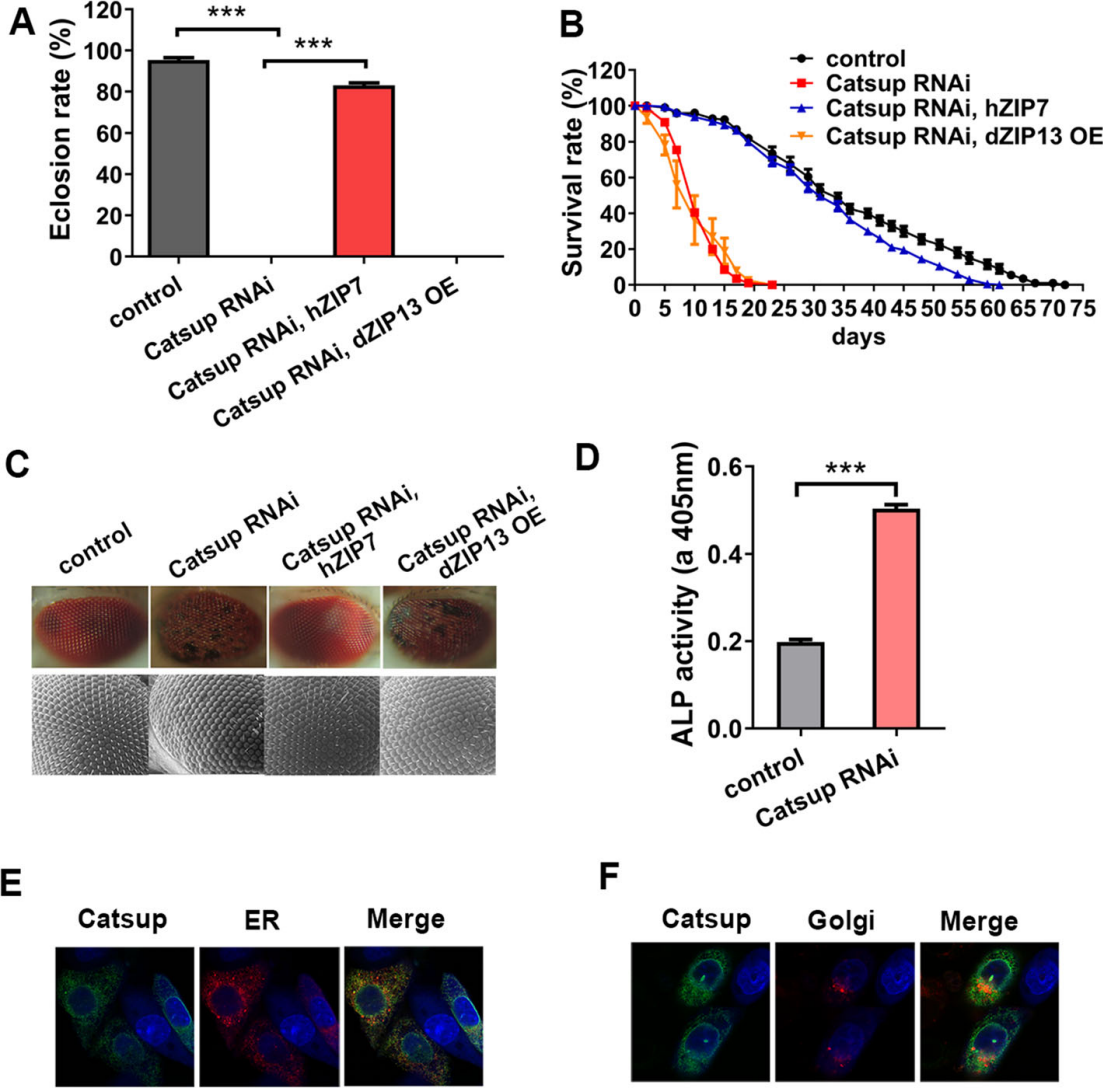
Catsup is the *Drosophila* ortholog of hZIP7 and functions as a zinc transporter. **A** The eclosion defect from ubiquitous *Catsup* RNA interference (*Da-Gal4*>*Catsup RNAi*) could be effectively rescued by hZIP7 expression, but not by dZIP13 OE. Genotypes of the flies used were *Da-Gal4> w*^*1118*^ (control); *Da-Gal4>Catsup RNAi*; *Da-Gal4>Catsup RNAi*, *hZIP7*; *Da-Gal4>Catsup RNAi*, *dZIP13 OE*. All values are presented as mean ± SEM; *n* = 6. ***p < 0.001. **B** The shortened lifespan of *Catsup RNAi* in the central nervous system was rescued by *hZIP7*, but not *dZIP13* OE. Genotypes of the flies used were *Elav-Gal4> w*^*1118*^ (control); *Elav-Gal4>Catsup RNAi*; *Elav-Gal4>Catsup RNAi*, *hZIP7*; *Elav-Gal4>Catsup RNAi*, *dZIP13 OE*. *n* = 6.**C** *hZIP7*, but not d*ZIP13 OE*, restored the eye blackspots of *Catsup RNAi* flies. Genotypes of the flies used were *Elav-Gal4> w*^*1118*^ (control); *Elav-Gal4>Catsup RNAi;* *El*av-*Gal4*>*Catsup RNAi*, *hZIP7*; *Elav-Gal4>Catsup RNAi*, *dZIP13 OE. n ≥ 6.* **D** The ALP activity, indicated by absorbance at 405 nm, was increased in the heads of *Catsup* RNAi flies when driven by the pan-neuronal driver *Elav-Gal4*, suggesting that *Catsup* RNAi in neurons led to zinc elevation in the Golgi. Genotypes of the flies used were *Elav-Gal4> w*^*1118*^ (control) and *Elav-Gal4>Catsup RNAi*. **E**, **F** The localization of Catsup in Chinese hamster ovary (CHOK1) cells. Catsup-FLAG was detected by anti-FLAG antibody. This immunofluorescence staining showed that Catsup partially co-localizes with the ER/Golgi in CHOK1 cells

There are at least ten putative ZIPs in the *D. melanogaster* genome [30–33]. Among them, *Catsup/CG10449* shares the highest overall homology with human *ZIP7* (hZIP7, also known as SLC39A7, 52% identity and 64% similarity), which functions as a zinc transporter located on the ER/Golgi [26]. Under normal dietary conditions, ubiquitous RNAi of *Catsup* by daughterless *Da-Gal4* produced developmental arrest at the embryonic stage (0% eclosion rate) (Fig. 1A). This eclosion defect resulting from *Catsup* knockdown could be effectively rescued by hZIP7 from ∼ 0 to ∼ 83% (Fig. 1A). As a control, expression of *dZIP13/CG7816* (also named *Zip99C*), a close homolog of hZIP7 but previously shown as an iron transporter located on the ER/Golgi [30], had no rescuing effect on *Catsup* RNAi (Fig. 1A). When *Catsup* was knocked down in the central nervous system (CNS), directed by the pan-neuronal *Elav-Gal4* [34], the flies exhibited early adult death or much reduced lifespan at 25 °C (Fig. 1B). The effects of hZIP7 and dZIP13 overexpression (OE) on the lifespan of *Catsup* RNAi were also analyzed. hZIP7 exhibited a strong rescuing effect, from ~ 8 to ~ 32 days, on the medium lifespans of *Catsup* RNAi flies raised at 25 °C, whereas dZIP13 OE had no effect (Fig. 1B). When *Catsup* was knocked down with *Elav-Gal4*, blackspots (cell death) appeared in the eyes (Fig. 1C). Consistent with the results described above, the blackspots were also markedly rescued by hZIP7 but not dZIP13 OE (Fig. 1C). The secretory enzyme alkaline phosphatase (ALP) activity is dependent on zinc loading in the Golgi, so its activity is responsive to zinc [35, 36]. In order to test whether zinc level was affected by *Catsup* in neurons, we tested the ALP activity of *Catsup* RNAi flies driven by *Elav-Gal4* (Fig. 1D). The results indicated that *Catsup* RNAi in neurons led to zinc elevation in the Golgi.

To examine the intracellular position of Catsup, a C-terminal flag-tagged Catsup was introduced into the Chinese hamster ovary (CHO-K1) cells. Immunofluorescence staining indicated that Catsup partially co-localizes with the ER/Golgi markers in CHO-K1 cells (Fig. 1E, F), consistent with the reported mammalian ZIP7 subcellular location [26]. Previous fluorescence imaging with the zinc indicator Zinpyr-1 has shown decreased zinc accumulation in the Golgi compartment in CHO-K1 cells expressing Catsup [37]. Based on the above results, that is, sequence homology, functional substitution, and subcellular localization, as well as roles in subcellular zinc distribution, it is reasonable to conclude that Catsup, like hZIP7, is a zinc transporter that mediates the transport of zinc from the ER/Golgi apparatus to the cytoplasm of the cell.

### Catsup loss results in detrimental organellar zinc accumulation and cytosolic zinc reduction

**Fig. 2:**
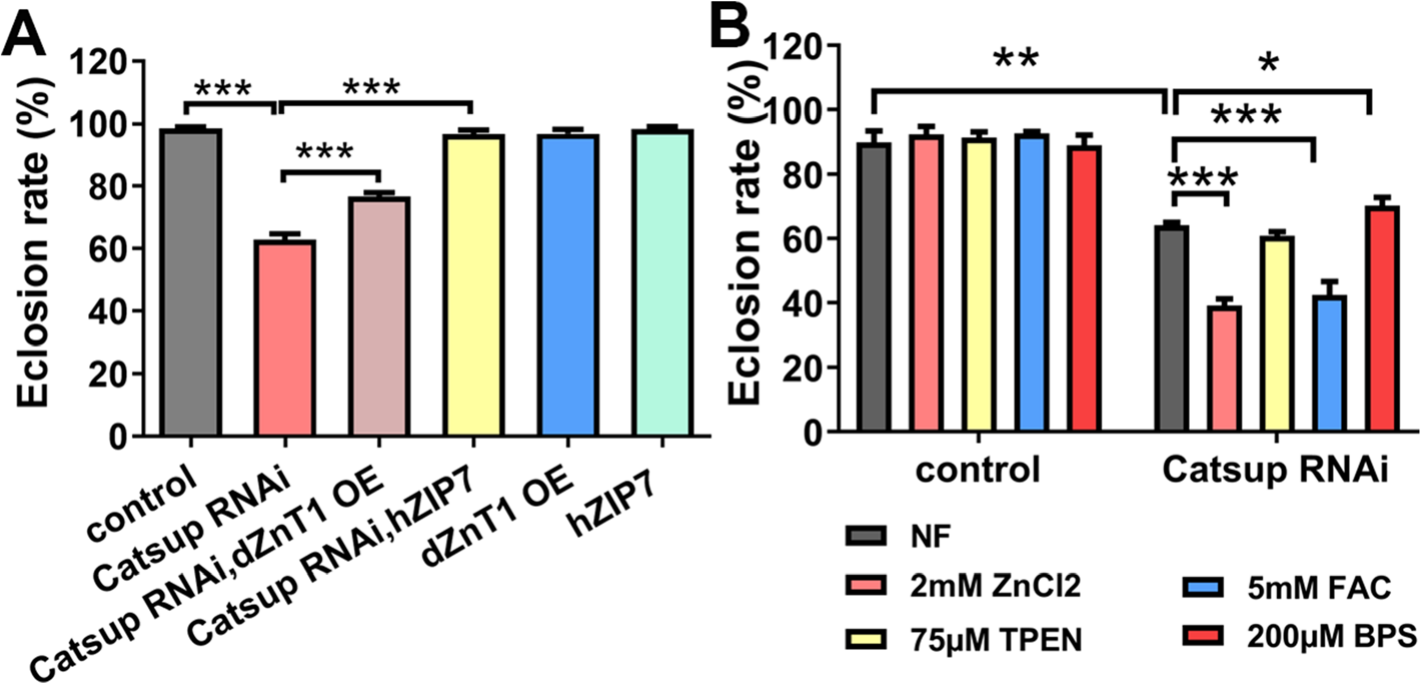
*Catsup* knockdown in dopaminergic neurons results in not only zinc but also iron sensitivities due to compartmentalized zinc toxicity. **A** The decreased eclosion defect of *Catsup RNAi* flies could be significantly rescued by hZIP7 or dZnT1 OE. Genotypes of the flies used were *TH-Gal4*> *w*^*1118*^ (control); *TH-Gal4*>*Catsup RNAi*; *TH-Gal4*>*Catsup RNAi*, *dZnT1* OE; *TH-Gal4*>*Catsup RNAi*, *hZIP7*. All values are presented as mean ± SEM; *n* = 6. ***p < 0.001. **B** The decreased eclosion defect of *Catsup RNAi* flies could be significantly influenced by dietary zinc or iron. The eclosion defect became deteriorated after ZnCl_2_ and FAC supplementation. Genotypes of the flies used were *TH-Gal4*> *w*^*1118*^ (control) and *TH-Gal4*>*Catsup RNAi*. All values are presented as mean ± SEM; *n* = 6. *p < 0.05, **p < 0.01, ***p < 0.001

Catsup functions as a transporter that redistributes zinc from the ER/Golgi to the cytosol. While Catsup reduction is associated with increased DA production, more severe loss of Catsup activity is lethal to the larvae [14]. In fact, ubiquitous *Catsup* knockdown produced no adult flies (Fig. 1B), and strong loss-of-function *Castup* mutant alleles have to be maintained in the heterozygous state with the wild-type allele [19]. This lethality could arise, directly or indirectly, from zinc dyshomeostasis either from zinc accumulation in the ER/Golgi and other organelles where Catsup is likely located, or zinc deficiency in the cytosol. In order to distinguish these two possibilities, we checked whether the phenotypes of *Catsup* RNAi could be rescued or aggravated by other zinc transporters. *Catsup* was specifically knocked down in the dopaminergic cells with the *TH-Gal4* driver [38]. Approximately 63% of these *Catsup* RNAi flies were able to eclose compared to the control (near 100%), and their lethality could be rescued by hZIP7 (Fig. 2A). If *Castup* RNAi lethality had been due to cytosolic zinc reduction, we expected that overexpression of* dZnT1/CG17723* (also named ZnT63C) [39, 40], a well-established plasma membrane zinc transporter, would exacerbate the toxicity of *Catsup* knockdown. Contrary to this notion, *dZnT1* OE, which should reduce intracellular zinc level, in fact alleviated the lethality of *Catsup* RNAi (Fig. 2A). As controls, the eclosion of *dZnT1* OE or hZIP7 driven by *TH-Gal4* showed no defects (Fig. 2A). This suggests that the death from *Catsup* RNAi came from zinc toxicity, i.e., compartmental zinc accumulation or associated processes such as trafficking in the secretory pathway. Indeed, ER stress has been well noted in previous studies after hZIP7 loss [24, 41].

The sensitivity to zinc of *Catsup* knockdown (*TH-Gal4*>*Catsup RNAi*) was further confirmed with a dietary zinc supplement. Supplementation of additional ZnCl_2_ significantly worsened the survival defect from 64 to 39% (Fig. 2B).

### TH activity enhancement in *Catsup* mutants arises from cytosolic zinc reduction instead of organelle zinc stress

**Fig. 3:**
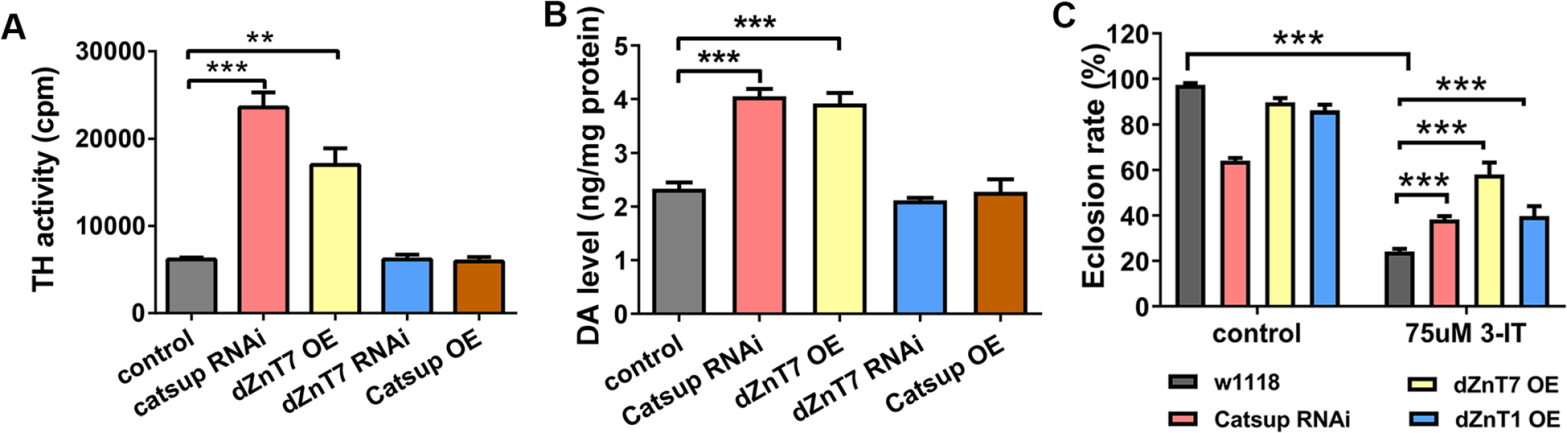
TH activity elevation in *Catsup* RNAi is due to cytosolic zinc reduction. **A** Either *Catsup RNAi* or *dZnT7* OE in the dopaminergic neurons significantly elevated TH activity. Genotypes of the flies used were *TH-Gal4*>*w*^*1118*^ (control), *TH-Gal4*>*Catsup RNAi*, *TH-Gal4*>*dZnT7 OE*, *TH-Gal4*>*dZnT7 RNAi*, and *TH-Gal4*>*Catsup OE*. All values are presented as mean ± SEM and included in Additional file 2: table S1; *n* = 4. **p < 0.01, ***p < 0.001. **B** DA level was elevated in *Catsup RNAi* and *dZnT7 OE* fly heads. Genotypes of the flies used were *TH-Gal4*>*w*^*1118*^ (control), *TH-Gal4*>*Catsup* RNAi, *TH-Gal4*>*dZnT7 OE*, *TH-Gal4*>*dZnT7 RNAi*, and *TH-Gal4*>*Catsup OE*. All values are presented as mean ± SEM and included in Additional file 2: table S2; *n ≥* 3. ***p < 0.001. **C** Expression of zinc exporters enabled resistance to 3-IT, a dopamine synthesis inhibiter. Genotypes of the flies used were *TH-Gal4*>*w*^*1118*^ (control), *TH-Gal4*>*Catsup RNAi*, *TH-Gal4*>*dZnT7 OE*, and *TH-Gal4*>*dZnT1 OE*. All values are presented as mean ± SEM; *n* = 6. *p < 0.05, **p < 0.01, ***p < 0.001

Catsup is responsible for intracellular zinc redistribution between the cytosol and the ER/Golgi. As mentioned, the disturbance of this distribution causes lethality as well as elevated DA production. Zinc accumulation in the secretory compartments is the basis for lethality. But what causes the DA increase? Is the observed elevated TH activity due to zinc elevation in the secretory compartments, or zinc reduction in the cytosol, or both? We first confirmed that TH activity is indeed modulated by redistributing intracellular zinc. This is meaningful since it could be argued that Catsup might be associated with some unidentified novel property other than zinc transport. dZnT7 is a well-characterized ER/Golgi-resident zinc transporter which functions in the opposite direction to Catsup [42]. *Catsup* RNAi and *dZnT7* OE both reduce cytosolic zinc and increase ER/Golgi zinc levels. When driven with the dopaminergic neuron driver *TH-Gal4*, *Catsup* RNAi and *dZnT7* OE exhibited respectively ∼300% and 200% more TH activity in the head, as compared to the control (Fig. 3A). Consistently, DA levels in the *Catsup* RNAi and *dZnT7* OE fly heads increased nearly 100% as compared to the control (Fig. 3B). This further strengthens the conclusion that increased TH activity in *Catsup* mutant flies is due to zinc dyshomeostasis, instead of some possible novel property other than zinc transport associated with dZIP7 (Catsup).

We then asked whether the zinc accumulation in the secretory compartments or the accompanied cytosolic zinc reduction would enhance DA synthesis. It is known that the lack of DA would lead to death in the flies. Indeed, when the fly was fed with the TH inhibitor 3-iodotyrosine (3-IT), the endogenous DA pool was significantly depleted [20] and the viability strongly suppressed (Fig. 3C). As expected, *Catsup* RNAi and *dZnT7* OE significantly rescued the viability (Fig. 3C). If the effect of *Catsup* on TH is through cytosolic zinc reduction, we would expect that the expression of the plasma membrane-resident zinc exporter dZnT1 also confers a rescue. Consistent with this notion, dZnT1 OE in the brain enabled more survivals under 3-IT treatment (Fig. 3C). This argues against the model that zinc accumulation in the ER/Golgi enhanced TH activity.

### *Catsup* loss may affect TH activity independent of TH phosphorylation and BH_4_ synthesis

**Fig. 4:**
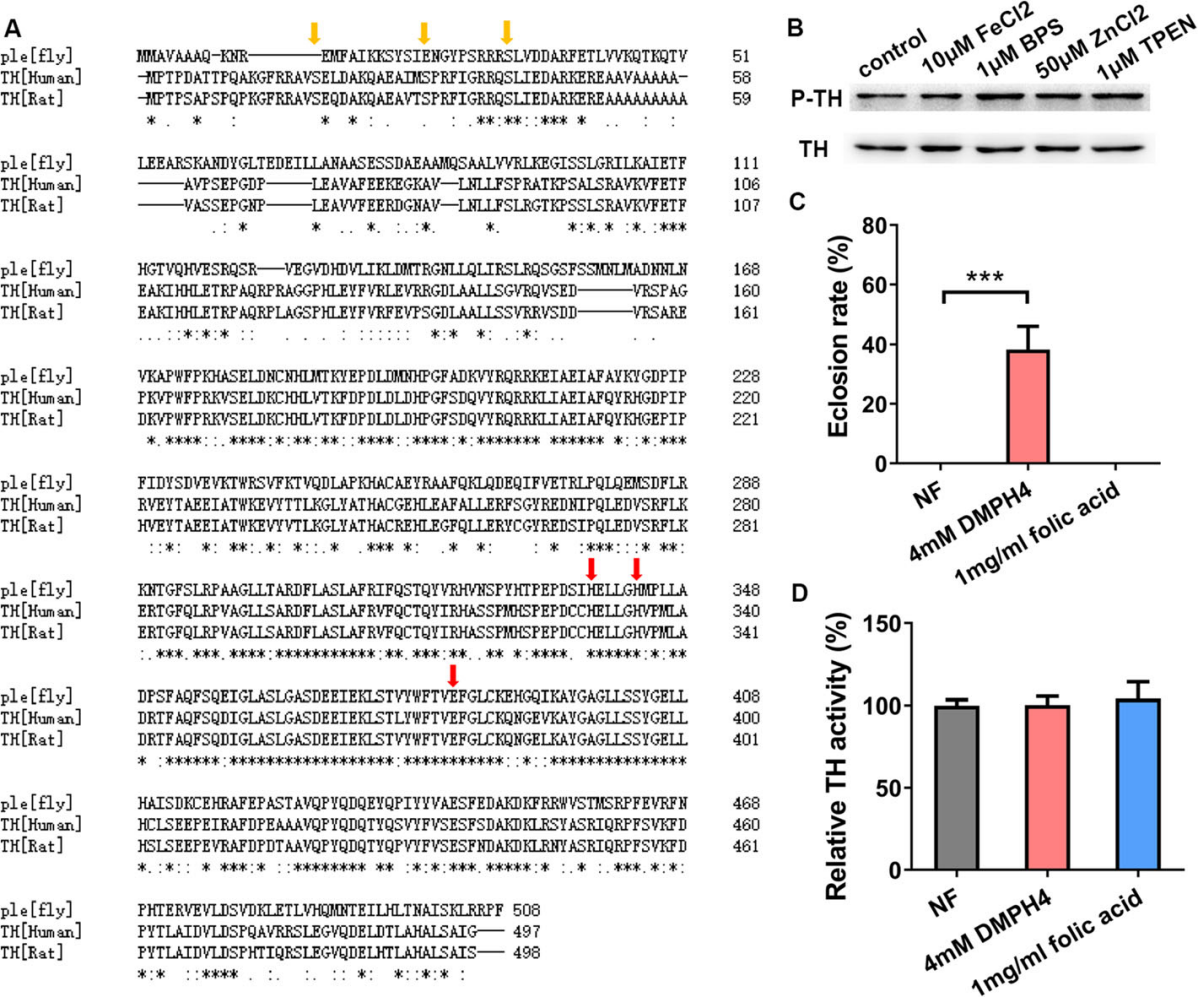
The in vivo effects of Catsup on TH appear primarily not due to TH phosphorylation and BH_4_ synthesis. **A** Sequence comparison among the fruit fly, human, and rat THs. Phosphorylation positions in mammalian TH and their corresponding amino acids in *Drosophila* TH are indicated with yellow arrowheads. Only the critical third phosphorylation site (Ser40) is conserved in the fly. Predicted metal-binding residues are marked with red arrows. **B** TH phosphorylation in SH-SY5Y cells under different metal or metal chelator treatments. No obvious and consistent changes were observed. An anti-phosphorylated TH antibody was used. Actin was used as a loading control. **C** The eclosion defect of *Da-Gal4>GTPCH RNAi* flies was rescued by DMPH_4_ supplementation in the food. Genotypes of the flies used were *Da-Gal4*>*GTPCH RNAi*. All values are presented as mean ± SEM; *n* = 6. ***p < 0.001. **D** DMPH_4_ supplementation in the food had no effect on the activity of TH. Genotype of the flies used was *w*^*1118*^. All values are presented as mean ± SEM; *n* = 6

Protein phosphorylation is one of the major mechanisms so far reported regulating TH activity [43]. *Drosophila* TH is encoded by the gene named *pale* [44, 45]. Bioinformatics analysis showed that *Drosophila* TH is similar to human TH [44, 46], sharing 54% identity and 74% similarity (Fig. 4A). The lack of commercial antibodies able to recognize phosphorylated *Droso*phila TH, so we tested in human neuroblastoma cells (SH-SY5Y cells) the effect of metals on mammalian TH phosphorylation at Ser40, the major player on TH activity among the three phosphorylation sites and the only one conserved between the fly and mammals [43, 47]. Western analysis indicated that the phosphorylation of TH at Ser40 was not prominently and consistently affected by zinc, iron, and chelators (Fig. 4B, Additional file 1: Figure S1 and Additional file 1: Figure S6). In other words, the regulatory effect of zinc or iron on TH activity may not be attributed to changes in phosphorylation.

TH requires tetrahydrobiopterin (BH_4_) as a cofactor for its activity [48]. *Catsup* mutations resulted in strongly elevated BH_4_ levels [21]. So, we wondered if the effect of zinc on TH activity is via its influence on BH_4_. The enzyme GTP cyclohydrolase I (GTPCH) is the rate-limiting enzyme for BH_4_ biosynthesis [21]*.* In *Drosophila*, GTP cyclohydrolase I (GTPCH) is encoded by *Punch* (*Pu*) [49]. Ubiquitous RNAi of *Pu* by *Da-Gal4* led to eclosion defects in *Drosophila*, which could be rescued by supplement of 6, 7-dimethyl 5, 6, 7, 8-tetrahydropterin (DMPH_4_), a synthetic cofactor for TH similar to but more stable than the natural cofactor (6R)-5,6,7,8-tetrahydrobiopterin (BH_4_) (Fig. 4C) [19, 50]. This indicates that dietary DMPH_4_ is physiologically accessible to TH in vivo. Consistently, it is also reported that pre-feeding flies with BH_4_ (0.34 mg/mL) could inhibit the firing of the NPF neurons [51]. Nevertheless, for normal flies reared on normal diet, we found that TH activity remained unaffected with additional BH_4_ supplementation (Fig. 4D). We consider this negative finding supporting the hypothesis that the amount of endogenous BH_4_ is probably saturated for the activation of TH under normal conditions, and additional BH_4_ may not further improve the TH enzyme activity. In the case of *Catsup* mutation, although TH activity is elevated, TH protein level is not [19]. This suggests that the observed increase in TH activity in *Catsup* loss flies may at least partially come from another route different from the increased BH_4_ synthesis.

### Zinc and iron antagonize with each other and directly impact TH activity

Based on our in vivo work as described above, we hypothesized an interaction between zinc and iron in regulating TH activity. Literature search revealed that it had previously been shown with in vitro biochemistry experiments that iron has a positive effect, while other divalent metal ions such as zinc, cobalt, and nickel ions have negative effects on recombinant human TH activity [52, 53]. However, it is not known whether these metal ions are relevant in vivo in regulating the activity of TH enzyme, especially when considering that intracellular labile metal ions are normally present at very low concentrations.

**Fig. 5:**
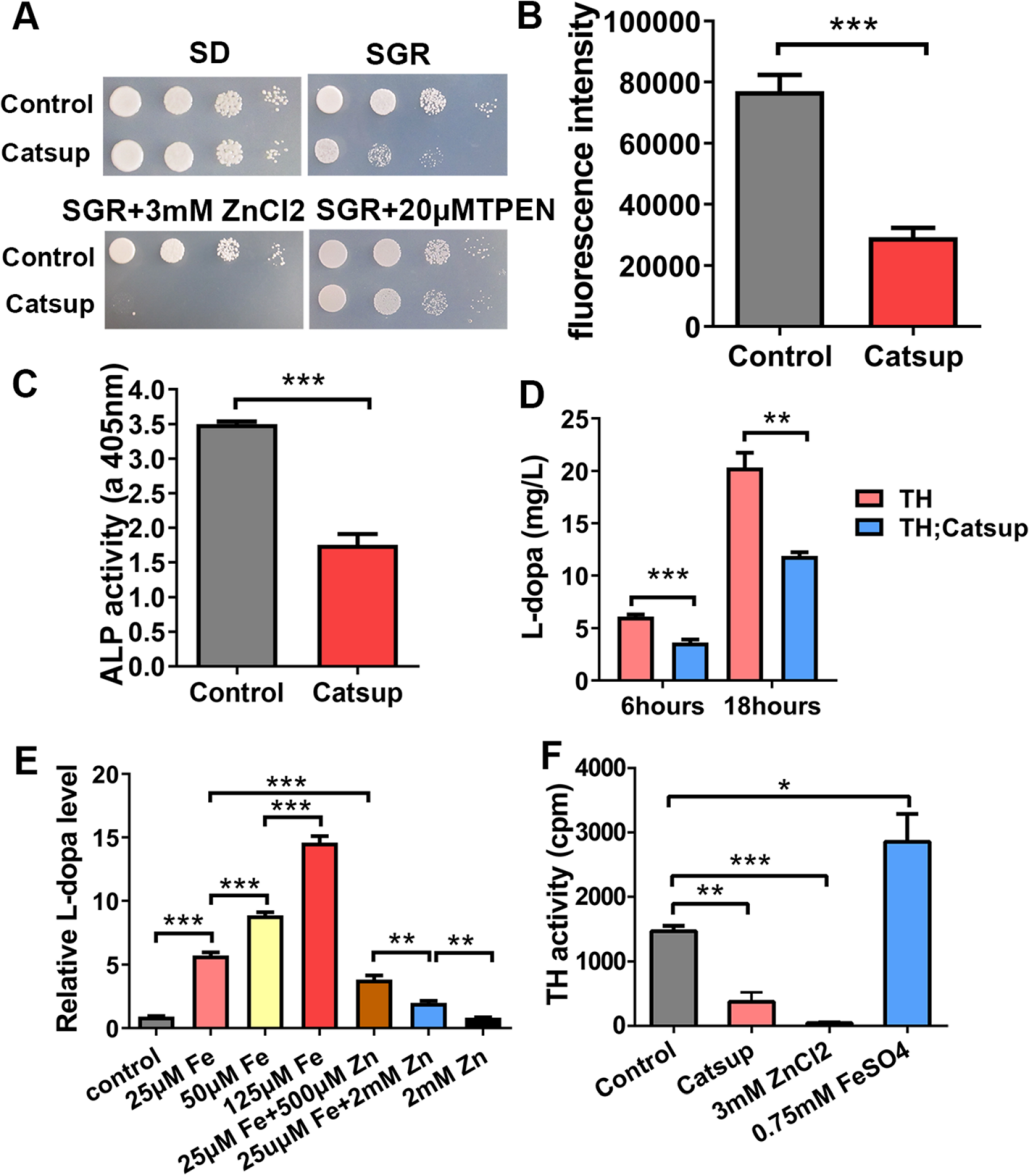
Zinc and iron potently but oppositely affect TH activity when expressed and assayed in yeast. **A** The growth defect of ZHY3 yeast expressing Catsup is exacerbated by zinc while rescued by TPEN. Transgenes could be induced on the synthetic galactose and raffinose (SGR) media but not on the synthetic dextrose (SD) media. *n* = 6. **B** The promoter activity of *ZRT1* is decreased when *Catsup* was expressed in ZHY3 yeast. All values are presented as mean ± SEM and included in Additional file 2: table S3; *n* = 6.***p < 0.001. **C** The ALP activity was reduced when Catsup was expressed in ZHY3 yeast. All values are presented as mean ± SEM and included in Additional file 2: table S4; *n* = 6. ***p < 0.001. **D** L-dopa production in the TH-expressing ZHY3 yeast could be inhibited by Catsup expression. Six hours and 18 h refer to the yeast incubation time for L-dopa production. All values are presented as mean ± SEM and included in Additional file 2: table S5; *n* = 4. **p < 0.01,***p < 0.001. **E** The generation of L-dopa in ZHY3 was elevated by iron and inhibited by zinc added in the media. All values are presented as mean ± SEM and included in Additional file 2: table S6; *n* = 3. **p < 0.01,***p < 0.001. **F** The activity of TH in ZHY3 was inhibited by Catsup expression or zinc while elevated by iron. All values are presented as mean ± SEM and included in Additional file 2: table S7; *n* = 3. *p < 0.05, **p < 0.01,***p < 0.001

We hypothesized that zinc and iron may confer direct and opposite effects on TH within a physiological context. TH is a cytosolic enzyme. From this perspective, the notion that the effect of *Catsup* mutation on TH arises from its cytosolic zinc reduction is reasonable. To formally investigate this possibility and exclude other likely interpretations, Catsup was heterologously expressed in yeast cells, a foreign, arguably relatively clean, and physiologically relevant system. Native yeast lacks the TH counterpart as well as BH_4_ and DA synthesis. We reasoned that if zinc and iron could impact TH independent of phosphorylation or BH_4_ variations, we might be able to detect metal-modulated TH activities in the yeast cells. We first tested whether Catsup indeed functions as a zinc transporter in the yeast. To do that, the ZHY3 strain lacking the Zrt1 and Zrt2 proteins for zinc uptake was used [54]. We considered that by using ZHY3 strain, a zinc-deficient cellular state would be created, and Catsup expression might be able to alleviate this cytosolic zinc deficiency. Interestingly, on the normal media ZHY3 with induced Catsup expression could not grow well (Fig. 5A). This defect could be attributed either to higher zinc in the cytosol or lower zinc in the secretory compartment. However, growth was restored with the cell-permeable zinc chelator TPEN, suggesting the defect arose from zinc accumulation in the cytosol. Consistently, *Catsup* expression rendered ZHY3 overtly sensitive to zinc stresses (Fig. 5A). These results are consistent with the expected role of Castup in redistributing zinc between the cytosol and the secretory compartments, only the stress in this case primarily arising from zinc accumulation in the cytosol in the presence of toxic amounts of zinc. As an alternative measurement, we used zinc-responsive *Zrt1*-luciferase reporter to indicate the cytoplasmic zinc pool. Double knockout of *Zrt1* and *Zrt2* zinc transporter genes results in a low cytoplasmic zinc pool in the yeast cell. When the cytoplasmic zinc pool is low, the transcriptional activity of *Zrt1* is upregulated, resulting in a higher luciferase activity. The expression of Catsup in ZHY3 led to a 60% decrease of luciferase activity compared with that in the control, suggesting an increased zinc level in the cytoplasm (Fig. 5B). The activity of ALP is very sensitive to zinc deficiency [55]. As expected, Catsup expression in ZHY3 significantly reduced the activity of ALP, indicating a decreased zinc level in the secretory pathway (Fig. 5C).

To analyze zinc’s effect on the activity of TH in yeast, *Drosophila* TH was then introduced into the ZHY3 strain. The yeast was incubated in the presence of 1 mM DMPH_4_ as yeast lacks endogenous BH_4_ synthesis. As shown in Fig. 5D, ZHY3 expressing TH synthesized L-dopa, and the expression of Catsup in ZHY3 resulted in a 40% decrease of the L-dopa level. We then tested the influence of exogeneous zinc and iron on the TH activity by measuring L-dopa production (Fig. 5E). The amount of L-dopa synthesized in ZHY3 expressing TH increased with iron and decreased with zinc supplementation (Fig. 5E). We also directly measured the TH activity with the radioisotope (Fig. 5F)*.* The results indicated that the TH activity was inhibited by Catsup and zinc, whereas induced by iron (Fig. 5F)*.* Given the unexpected growth inhibitory effect of Catsup on ZHY3, the effect of zinc and iron on L-dopa synthesis was further examined with wild-type INVSc1 strain expressing TH, and similar results were obtained (Additional file 1: Figure S2). These results indicated that the activity of TH heterologously expressed in the yeast can be influenced by the intracellular iron or zinc available to cells. Noteworthy is that since the yeast is unable to synthesize BH_4_, the fact that under a fixed BH_4_ level zinc and iron could still markedly affect TH activity indicates that the zinc and iron effect on TH could be direct.

**Fig. 6:**
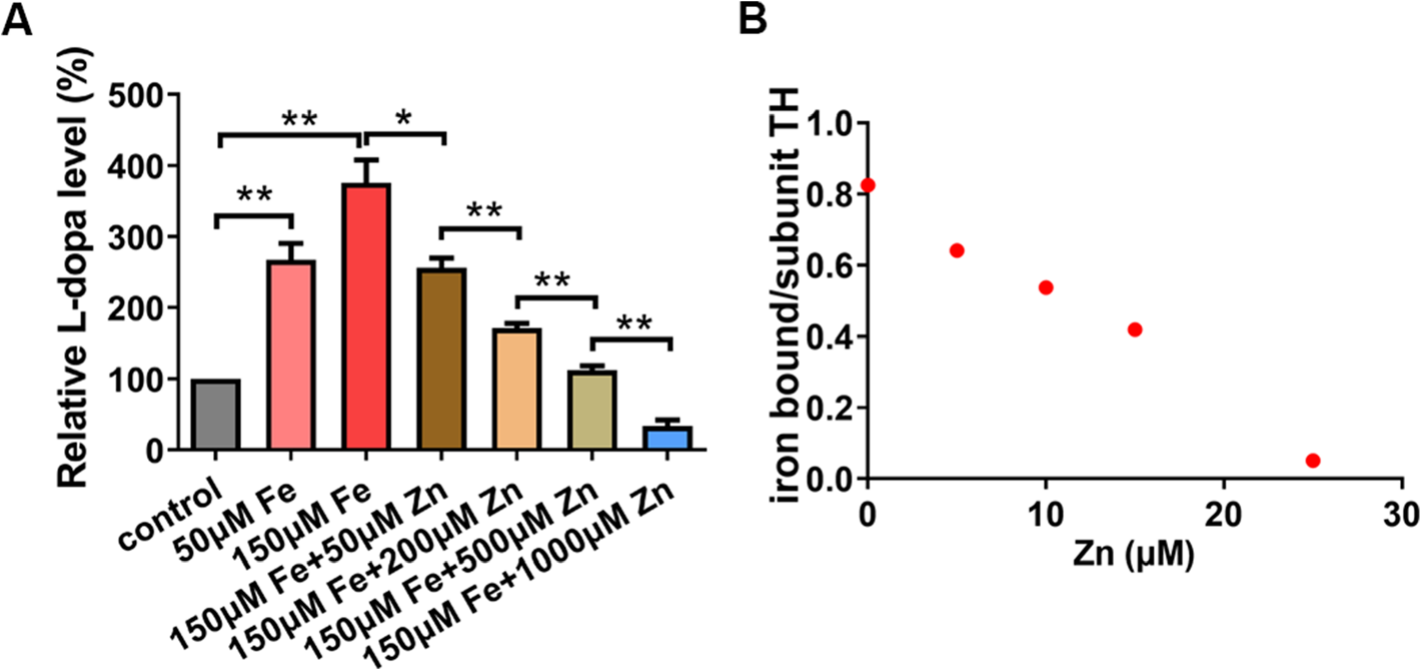
Zinc ion interferes with ferrous iron binding to TH. **A** The production of L-dopa in *E. coli*-expressing TH was elevated by iron and inhibited by zinc added in the media. All values are presented as mean ± SEM and included in Additional file 2: table S8; *n* = 3. *p < 0.05, **p < 0.01. **B** Binding of iron to *Drosophila* TH. Apo-TH was incubated with 15 μM ferrous iron and different concentrations of zinc. Free and bound metal ions were separated by rapid ultrafiltration, and the iron level was determined by BPS-based colorimetric assays. The amount of bound iron decreased with increased zinc concentration in the buffer. All values presented are included in Additional file 2: table S12

We also tested TH responsiveness to iron and zinc in *E. coli*. Native *E. coli* also lacks TH and DA synthesis, as well as the essential cofactor BH_4_. *Drosophila* TH was expressed in *E. coli* and provided with exogenous DMPH_4_. As shown in Fig. 6A, *E. coli*-expressing *Drosophila TH* synthesized L-dopa, and the amount of L-dopa synthesized increased with incremental elevations of iron and decreased with zinc. The expression of TH, on the other hand, appeared not much affected by the addition of zinc and iron (Additional file 1: Figure S3). Therefore, both the experiments in the yeast and *E. coli* indicate that TH activities within a cell could be modulated robustly and possibly directly by iron and zinc homeostasis, and that iron and zinc antagonize each other in regulating TH activity.

It is known that iron is a cofactor of TH and the redox state of the iron is essential for its function [52]. Site-directed mutagenesis studies and crystal structure analysis revealed that the side chains of two histidine residues and one glutamate residue in the active site of mammalian TH are directly involved in iron coordination [56]. Some other divalent metal ions also could bind with TH and inhibit its activity in vitro [52, 53]. Not surprisingly, *Drosophila* TH and human TH are well conserved in these iron-binding sites (Fig. 4A). We hypothesized zinc could compete with iron binding with *Drosophila* TH and subsequently inhibit its activity. To this end, we performed a metal-binding assay. *Drosophila* TH was expressed in *E. coli* and purified. The purified recombinant *Drosophila* TH was incubated with Fe^2+^ and Zn^2+^. The bound and free iron fractions were then separated and measured by BPS-based colorimetric assays (Fig. 6B). We observed that *Drosophila* TH contained about 0.8 iron atom/subunit when incubated with ferrous iron, and the amount of bound iron decreased with increasing concentrations of zinc in the incubation buffer (Fig. 6B). The competing binding of iron and zinc on TH was further confirmed by ICP-MS (Additional file 1: Figure S4). Notably, zinc appears to be able to bind TH at multiple sites, likely as a result of non-specific binding in particular at high concentrations. These results indicate that zinc regulates *Drosophila* TH activity by affecting TH binding with iron.

### Modulation of zinc and iron homeostasis alters phenotypes associated with PD

**Fig. 7:**
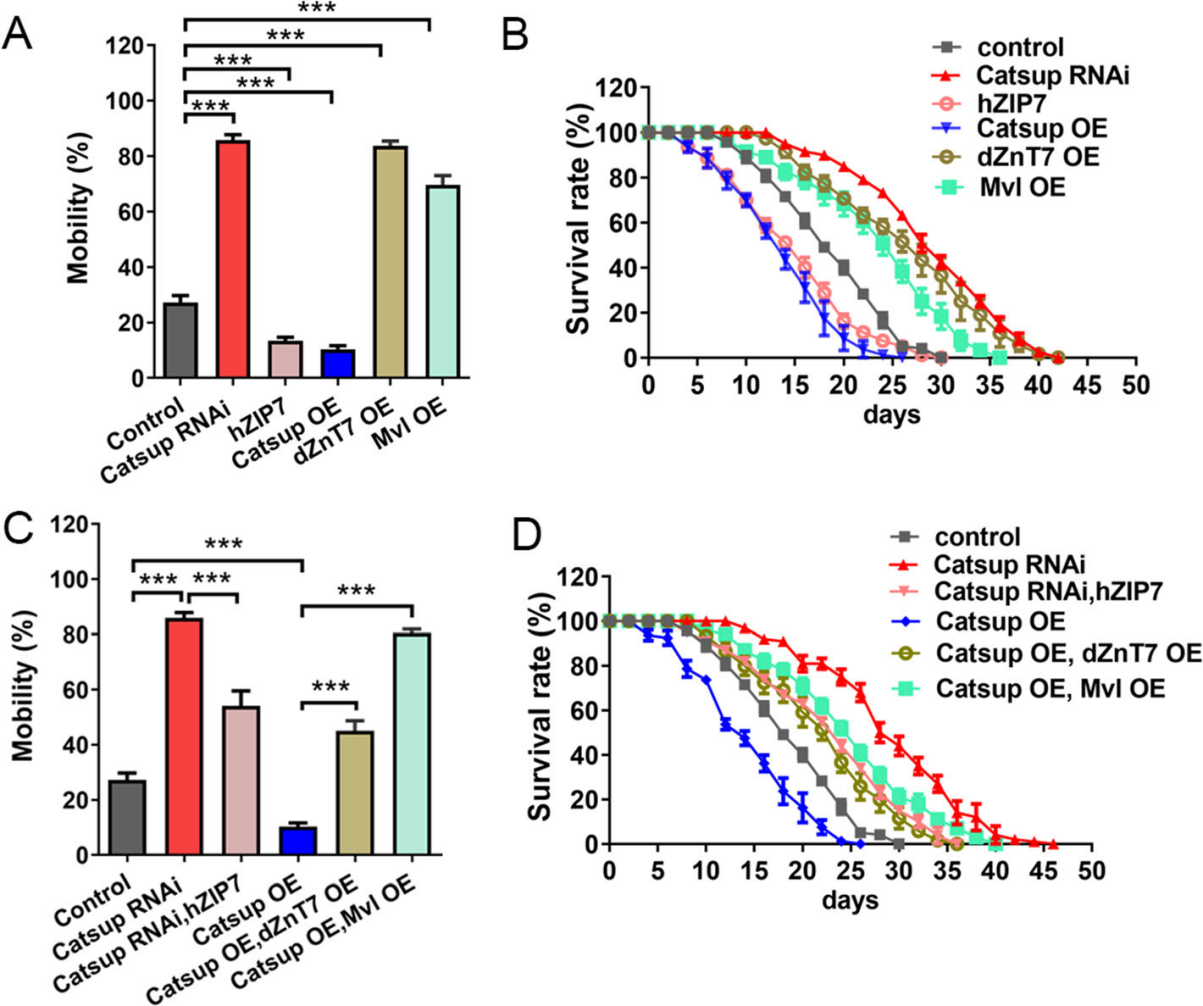
Modulation of zinc and iron homeostasis is an effective means to alter a spectrum of phenotypes associated with PD. **A** The mobility defect of PD flies (exposing to rotenone) could be rescued by *Catsup* RNAi, *dZnT7* OE, or *Mvl* OE. Genotypes of the flies used were *TH-Gal4*>*w*^*1118*^ (control), *TH-Gal4*>*Catsup RNAi*, *TH-Gal4*>*Catsup OE*, *TH-Gal4*>*dZnT7 OE*, and *TH-Gal4*>*Mvl OE*. All values are presented as mean ± SEM and included in Additional file 2: table S9; *n ≥ 6*. ***p < 0.001. **B** The shortened lifespan of PD flies (exposing to rotenone) could be modified by adjusting zinc transporter expressions. Genotypes of the flies used were *TH-Gal4*>*w*^*1118*^ (control), *TH-Gal4*>*Catsup RNAi*, *TH-Gal4*>*Catsup OE*, *TH-Gal4*>*dZnT7 OE*, and *TH-Gal4*>*Mvl OE*. **C** The mobility rescue effect of *Catsup RNAi* on PD flies (exposing to rotenone) could be neutralized by hZIP7 expression. The exacerbated defect of *Catsup OE* on PD flies could be rescued by *dZnT7* OE and *Mvl* OE. Genotypes of the flies used were *TH-Gal4*>*w*^*1118*^ (control), *TH-Gal4*>*Catsup RNAi*, *TH-Gal4*>*Catsup RNAi*, *hZIP7 OE*, *TH-Gal4*>*Catsup OE*, *TH-Gal4*> *Catsup OE*;*dZnT7 OE*, *TH-Gal4*> *Catsup OE*, and *Mvl OE*. All values are presented as mean ± SEM and included in Additional file 2: table S10; *n ≥ 6.*. ***p < 0.001. One-way analysis of variance (ANOVA) was used for multiple groups. **D** The lifespan rescue of *Catsup* RNAi on PD flies (exposing to rotenone) could be neutralized by hZIP7 expression. The exacerbated defect of *Catsup* OE on PD flies could be rescued by *dZnT7* OE and *Mvl* OE. Genotypes of the flies used were *TH-Gal4*>*w*^*1118*^ (control), *TH-Gal4*>*Catsup RNAi*, *TH-Gal4*>*Catsup RNAi*, *hZIP7 OE*, *TH-Gal4*>*Catsup OE*, *TH-Gal4*> *Catsup OE*, *dZnT7 OE*, *TH-Gal4*> *Catsup OE*, and *Mvl OE*. All values are presented as mean ± SEM and included in Additional file 2: table S11; *n ≥ 4*. ***p < 0.001

PD is a neurodegenerative disorder characterized in its late phase by sustained loss of dopaminergic neurons from the substantia nigra pars compacta (SNpc) and other brainstem regions [57]. Since DA loss is a cardinal feature of PD and L-dopa is utilized as an initial therapy to treat PD, we wondered whether the effect of zinc and iron on TH could influence PD phenotypes. Rotenone is a specific high-affinity inhibitor of mitochondrial complex I [58] and has been shown to be an environmental toxin (along with other chemicals such as MPTP and paraquat) contributing significantly towards the occurrence of sporadic PD in humans and animal models [59]. Rotenone exposure caused serious mobility impairment and reduced lifespan for *Drosophila* at 25 °C (Fig. 7A, B). When raised at 25 °C for 7 days in the presence of rotenone, about 30% of flies reached the subjectively designated height (8 cm) within a set time (7 s) (Fig. 7A). *Catsup* RNAi and *dZnT7* OE were significantly rescued, whereas *Catsup* OE exacerbated, the mobility impairment (Fig. 7A). Consistent with the results described above, *Catsup* RNAi and *dZnT7* OE extended the reduced longevity of rotenone-treated flies, whereas *Catsup* OE shortened it (Fig. 7 B). The overexpression of *Malvolio (Mvl)*, an iron importer which presumably increases the iron level in the cytoplasm, also exhibited dramatic rescuing effects on the mobility impairment and the reduced lifespan (Fig. 7A, B). *Catsup* RNAi, *dZnT7* OE, *Catsup* OE, and *Mvl* OE all exhibited mobility and survival defects when raised on normal food (Additional file 1: Figure S5). However, their effects on roteone-induced PD are diverse. *Catsup* OE, which on normal food survived better than *Catsup* RNAi and *dZnT7* OE (Additional file 1: Figure S5), faired much worse on rotenone (Fig. 7A, B). In fact, although *Catsup* RNAi and *dZnT7* OE were harmful on their own in the normal context (Additional file 1: Figure S5), they displayed a strong rescuing effect on rotenone-treated flies (Fig. 7A, B).

Further study indicated that the rescue effect of *Catsup* RNAi on flies with rotenone exposure could be inhibited by *hZIP7* OE, and the exacerbation effect of *Catsup* OE on these flies could be rescued by dZnT7 OE and *Mvl* OE (Fig. 7C, D). Taken together, these results indicated that modulating the zinc and iron levels in the *Drosophila* brain could modify a whole range of symptoms of rotenone-treated flies.

## Discussion

Zinc and iron are critical to a variety of proteins and are essential for normal CNS development and function [60]. Previous studies showed that *Catsup* mutants have significantly elevated TH activity, resulting in abnormally high levels of catecholamines [19]. In this study, we confirmed that Catsup is indeed functionally analogous to hZIP7, transporting zinc from the secretory compartments into the cytoplasm. We found that manipulating *Catsup* in the brains can greatly modulate DA synthesis and PD progress by modulating TH activity through the interference of iron. We provided in vivo evidence that TH activity is affected by a balance of zinc and iron levels. Further study indicated that manipulation of zinc levels and iron levels in PD brains may be a novel therapeutic strategy. This may hold great significance for the prevention and treatment of PD and even other diseases caused by catecholamine dysfunction.

Ubiquitous manipulation of zinc and iron homeostasis often resulted in reduced viability. For this reason, dramatic modulation of metal homeostasis may not be feasible in PD rescue. We have primarily relied on genetic measures to modulate regional metal homeostasis such as in the brain or TH-positive cells to achieve a significant effect. It is envisioned that in clinical cases, targeted modification of metal homeostasis may be necessary to obtain satisfactory results. This is particularly so when the blood-brain barrier is taken into account, which may partially insulate the brain from the circulation system. Indeed, in our case, the nutritional supplement of metals in the food was much less efficient to reach the kind of rescue we saw with tissue-specific genetic interventions.

Catsup is a member of the ZIP (Slc39A) transporter family [30]. Most ZIP family members transport zinc, but a few have been found to transport other metals, including ZIP8, ZIP13, and ZIP14 [61]. Catsup shares the highest overall homology with human ZIP7 [25, 30], which is an identified zinc transporter [26]. Simultaneously, Catsup also shares high homology with human dZIP13, which fulfills the iron effluxing role in *Drosophila* [30]. In this study, we studied the function of Catsup and verified that it is a zinc transporter located in the secretory compartments. In a previous study, we provided in vivo functional analysis to differentiate different transporting functions of these two ZIPs; the HNXXD motif is required for zinc transport activities for Catsup [37].

The activity of many enzymes is regulated by metal ions. For example, the activity of superoxide dismutase (SOD) can be induced by copper but inhibited by iron or cadmium [62]; cytochrome c oxidase needs iron and copper to execute its activity, but the presence of zinc inhibits its activity [63]. So, it seems important to balance these metal ions in vivo to regulate the activity of these enzymes. In this study, we showed that in vivo iron stimulates while zinc inhibits TH activity, consistent with the previous report that the activity of recombinant human TH was rapidly activated by incubation with iron and inhibited by some other metal ions in vitro [52].

Protein phosphorylation is reported as one major mechanism for controlling the activity of TH [43]. TH phosphorylation is regulated by several protein kinases and protein phosphatases [43]. It is well known that zinc plays an important role in affecting phosphorylation signaling [64, 65]. Here, we found that the TH phosphorylation is not appreciably affected by zinc or iron modulation, so the effect of Catsup on TH activity seems through a route other than phosphorylation.

**Fig. 8:**
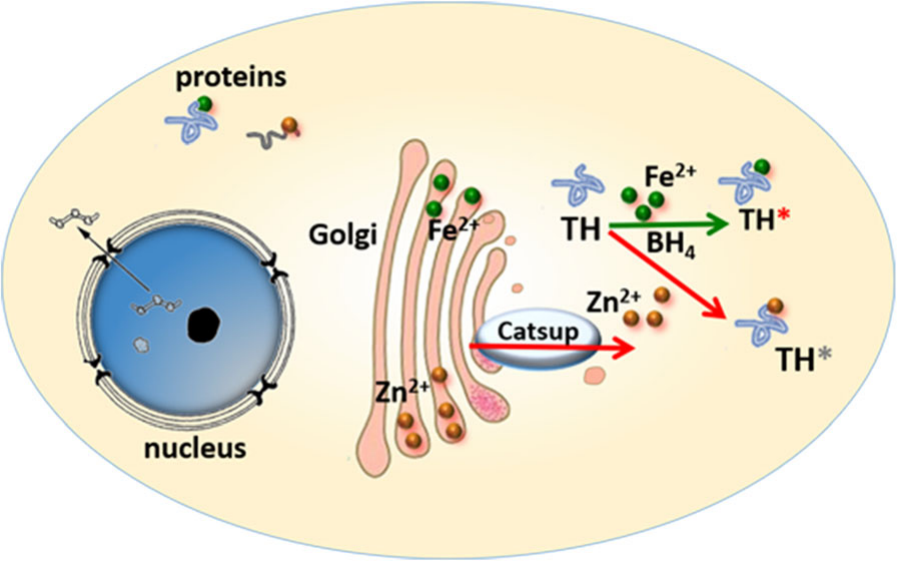
A model to explain Catsup’s effect on TH activity. Zinc appears to be an important regulator of TH activity or dopamine production in vivo. When *Catsup* is mutated, zinc fails to be effectively redistributed between ER/Golgi and cytosol, leading to zinc elevation in the secretory pathway but reduction in the cytosol. Zinc normally antagonizes iron in regulating TH activity. When zinc release is blocked, TH has better access to iron and its activity is elevated

TH requires BH_4_ as a cofactor [48], and the enzyme activity is catalyzed by ferrous iron [66]. As a result, the synthesis of catecholamines is not only dependent on TH activity but also on the level of BH_4_ and iron. Because the TH activity is not increased in flies feeding with DMPH_4_, we proposed that under normal conditions, the BH_4_ level in the body may be sufficient or saturated, so that excess BH_4_ in vivo has no further contribution to the TH activity. Expression of TH within the yeast and *E. coli*, both of which lack BH_4_ synthesis and likely some other regulatory pathways tuning TH activity, indicated that TH activity is robustly responsive to iron and zinc modulation. Altogether, we propose that iron in the cytoplasm binds TH to catalyze its activity, and zinc can compete with iron binding in TH. Since Catsup is responsible for zinc release from the secretary pathway to cytoplasm, *Catsup* mutations lead to zinc deficiency in the cytoplasm, and in consequence, more iron could bind to TH, resulting in the increased TH activity (Fig. 8).

Increasing amounts of evidence show that imbalance of trace metal homeostasis is strongly related to the pathological process of PD [67, 68]. Here, we revealed that there exists a new way to regulate DA synthesis in vivo through modulating metal homeostasis. The knowledge gained in this study appeared to be adaptable in ameliorating PD features in a fly model. It is reasonable to speculate that cells could physiologically modulate dopamine synthesis through zinc control, by maintaining a balance between zinc storage and release to adjust the cytosolic zinc levels. Indeed, mammalian ZIP7 was reported to be regulated by phosphorylation [69]. Therefore, it is expected that cells could adjust DA levels through modifying expression or posttranslational modifications of ZIP7 in response to physiological changes.

These results may suggest new strategies for the prevention and treatment of PD and some other diseases closely related to DA. Considering zinc depletion and iron increase may achieve similar end results, in our opinion, it may be preferable to use iron limitation strategy when depressing dopamine levels is intended or zinc limitation in the case of boosting dopamine production. This is because iron may in short term raise dopamine levels; it may also simultaneously stimulate elevated production of reactive oxygen species, heightening dopaminergic neuron damages in the long run. Besides PD, DA is closely related to pain, drug addiction, attention deficit hyperactivity disorder, and many kinds of mental diseases such as psychosis, schizophrenia, and depression [2, 5]. Unfortunately, the etiology of many of these diseases remains less well clarified. It would be interesting to see how the balance of zinc and iron could modulate the disease progression for some of these physiological disorders that might be DA-related.

## Conclusions

Analyses of dopamine elevation in *Catsup* mutation/knock-down revealed an important role of zinc homeostasis in regulating TH activity. Zinc antagonizes ferrous iron, a cofactor of the TH enzyme, to exert a negative effect on TH. This work enriches our understanding of dopamine regulation in vivo and may help our fight against PD and other forms of dopamine-related diseases.

## Methods

### *Drosophila* strains, yeast strains, and maintenance

*Drosophila* strains used in this study are listed as follows: *Da-Gal4* (Bloomington #8641), *Elav-Gal4* (Bloomington #458), and *TH-Gal4* (Bloomington #8848), all from the Bloomington *Drosophila* Stock Center. *p* *Catsup* RNAi (VDRC #100095) and dZnT7 RNAi (VDRC #107388) were obtained from the Vienna *Drosophila* RNAi Center. Transgenic fly strains used in this report have been previously described, including *dZIP13* OE [37], *dZIP1* OE [42], *dZnT1* OE [39]. pUAST-*Malvolio* construct (*Mvl* OE) was injected into *w*^*1118*^ background flies following standard protocols. One line with relatively more severe neuronal phenotype when directed by *ELav-Gal4* was chosen for the studies reported here.

All flies were maintained on standard cornmeal media at 25 °C with 60% humidity under a 12-h light–dark cycle unless otherwise stated. Concentrations of supplemented metals or metal chelators used were as follows: 5 mM ferric ammonium citrate (FAC; Sigma-Aldrich), 150 μM bathophenanthroline disulfonate (BPS; Sigma-Aldrich), 50 μM *N*,*N*,*N′*,*N′*-tetrakis (2-pyridylmethyl) ethylenediamine (TPEN; Sigma-Aldrich), and 2 mM ZnCl_2_ (Beijing Yili Fine Chemicals Ltd. Co., Beijing, China). For inhibitor studies, concentration of 3-Iodo-*L*-tyrosine (3-IT; Sigma-Aldrich) used was 75 μM. For the rotenone feeding experiment, 2-day-old adult flies were transferred to vials with instant *Drosophila* medium and exposed to 150 μM freshly prepared rotenone (Sigma-Aldrich) for 7 days. The medium was replaced every day to ensure that the rotenone was effective.

The ZHY3 strain lacking the Zrt1 and Zrt2 proteins for zinc uptake was used in this study [54].

### Cell culture and plasmid transfection

For subcellular localization experiments, CHO-K1 cells were maintained in DMEM (Invitrogen) containing 10% fetal bovine serum (FBS, Gibco BRL, Gaithersburg, MD, USA) at 37 °C under 5% CO_2_ atmosphere. When the cells reached 80% confluence, they were transfected with pIRESneo-Catsup-FLAG using Lipofectamine™ 3000 (Invitrogen) in accordance with the manufacturer’s instructions. After 24 h, cells were fixed, stained with the FLAG antibody (1:500) and the Golgi marker (anti-GM130, 1:500) or ER marker (anti-PDI, 1:500), and imaged with the ZEISS LSM780 microscope. Anti-FLAG mouse monoclonal antibody (ab18230), anti-FLAG rabbit polyclonal antibody (ab1162), anti-GM130 rabbit polyclonal antibody (ab30637), and anti-PDI mouse monoclonal antibody (ab2792) were obtained from Abcam (Cambridge, MA, USA). Secondary antibodies include cy3-conjugated goat anti-mouse and cy3-conjugated goat anti-rabbit IgG (Zhongshan Goldenbridge Biotechnology, Beijing, China). The experiments were repeated three times.

SH-SY5Y cells were used to test the effect of metals on mammalian TH phosphorylation at Ser40. SH-SY5Y cells were maintained in DMEM (Invitrogen) with 10% FBS and 1% PS under an atmosphere of 95% air and 5% CO_2_ at 37 °C. To test metals’ effects, 1 μM BPS, 50 μM ZnCl_2_, 1 μM TPEN, and 10 μM FeCl_2_ (2 mg/mL vitamin C were added in the medium to inhibit iron oxidation) were respectively added in the medium. The experiments were repeated three times.

### Eclosion, mobility, and longevity assays

For eclosion assays, Gal4 lines were crossed with various transgenic lines for ~ 3 days, as indicated in each experiment, and allowed to lay eggs on juice-agar plates for ~ 24 h. Newly hatched first-instar larvae were transferred to normal food or food supplemented with metals or metal chelators, as indicated. The density of each vial was controlled to 60–100 larvae, and the total number of emerging adults of each genotype was counted [30]. Assays were done with six replicates for each group, and the experiments were repeated at least three times. The results from all experiment runs were pooled together for analyses.

To assay the mobility of PD flies, 20 females were placed in a vertical glass vial. After a 1-h recovery from CO_2_ exposure, flies were gently tapped to the bottom of the column. The number of flies reaching the indicated height (~ 8 cm) was counted after 7 s of climbing under red light. Six parallel group tests were conducted for each genotype, and the experiments were repeated at least three times [34]. The presented data are from the results of all experiments.

For longevity assays, 3-day-old adult males or females were kept in vials (20 individuals of each vial) containing cornmeal medium and maintained at 25 °C. Flies were transferred to fresh media every 2–3 days, and the number of live flies was recorded every alternate day. Six parallel group tests were conducted for each genotype, and the experiments were repeated at least three times [34]. The data were presented as Kaplan–Meier survival distributions, and significance was determined by log-rank tests.

### Eye morphology analysis

To phenotype eye morphology, flies were collected after eclosion at 18 °C and frozen to death at − 80 °C for one night. The eyes were photographed using a Zeiss imager A1 stereomicroscope. More than six flies were scored per genotype, and each experiment was repeated three times. Scanning electron microscopy analysis of fly eyes was performed as described previously with FEI Quanta 200 [70].

### Alkaline phosphatase(ALP) activity assay

ALP activity assays were performed as described previously with some modifications [71]. For yeast samples, yeast was cultured in synthetic dextrose media (SD) at 30 °C to OD = 0.4, collected by centrifugation at 3000*g* for 5 min, and then transferred to the induction medium (synthetic glucose and raffinose (SGR) media). The yeast was cultured in SGR media at 30 °C to OD = 1, collected by centrifugation, and washed three times with ice-cold PBS. The collected yeast was homogenized in ALP lysis buffer (1.0 mM Tris–HCl pH 7.4, 0.5 mM MgCl_2_, and 0.1% Triton X-100), and the protein concentration was measured by the BCA kit (Thermal). Approximately 2 μg protein was added to 90 μL solution A (1.0 M diethanolamine, 0.5 mM MgCl_2_ pH 9.8) and 10 μL solution B (150 mM p-nitrophenyl phosphate). After incubation for 30 min at 30 °C, ALP activity was measured based on the p-nitrophenol release by its absorbance at 405 nm.

For fly samples, ∼ 80 adult heads were lysed in the ALP lysis buffer, then ∼2 μg protein was added to 90 μL solution A and 10 μL solution B. The absorbance at 405 nm was measured after incubation for 30 min at 25 °C. The experiments were repeated three times.

### Luciferase activity assay

Yeast was cultured in SD media at 30 °C to OD = 0.4, collected by centrifugation at 3000*g* for 5 min, and then transferred to SGR media. The yeast was cultured in SGR media at 30 °C to OD = 1, collected by centrifugation, and washed three times with ice-cold PBS. The collected yeast was homogenized in cold PBS, and the protein concentration was measured by the BCA kit (Thermal). Approximately 10 μg protein was used to test the luciferase activity by Firefly Luciferase Reporter Gene Assay Kit (Beyotime, #RG027); fluorescence intensity was measured for 10 s by the fluorescence microplate reader. In order to avoid the error caused by the difference in the amount of samples, the reporter gene of Renilla luciferase was used as an internal reference.

### Western blot analysis

SH-SY5Y cells were used to test the effect of metals on mammalian TH phosphorylation at Ser40. Antibodies of anti-tyrosine hydroxylase (AB152) and anti-tyrosine hydroxylase, phosphoSer 40 (AB5935) were purchased from Sigma-Aldrich (Shanghai, China). The secondary antibody HRP-conjugated goat anti-rabbit IgG was purchased from Zhongshan Goldenbridge Biotechnology (Beijing, China). For Western blot analysis, cells were homogenized in the buffer containing 1% Triton X-100 plus 10% proteinase inhibitor cocktail (Sigma), centrifuged, separated on 10% SDS-PAGE, and transferred to nitrocellulose membranes (Millipore, Watford, UK). Signals were developed with the ECL detection kit (Vigorous Biotechnology, Beijing, China). The experiments were repeated three times.

### Metal sensitivity/resistance assay in yeast

Catsup was cloned into pYES2 (Invitrogen) and transformed into ZHY3. The vector pYES2 was also transformed into yeast and used as the control. For growth testing on agar plates (spotting assay), yeast grown in the culture medium (synthetic dextrose media, SD) was 10-fold serially diluted with sterile ddH_2_O and then spotted on SD (the transgene could not be induced on this medium) or synthetic galactose and raffinose media (SGR, the transgene could be induced on this medium) plates with or without metal or chelators. Incubation was all at 30 °C; 3 mM ZnCl_2_ or 20 μΜ TPEN was added in the medium, *n* = 6.

### Metal ions binding assays of TH

A double-tagged *Drosophila* TH protein (dTH) expression plasmid containing N-terminal His6 and SUMO tag (pET28a-His6-SUMO-dTH) was constructed. The ensuing plasmid was transformed into *E. coli* BL21(DE3). Protein expression was induced by 250 μM β-D-1-thiogalactopyranoside (IPTG) with 100 μM FeSO_4_ supplied in LB medium for enzyme stability. Induction proceeded at 16 °C for 14 h. The His6-SUMO-dTH protein was purified with Ni-NTA resin, and the tags were removed by ULP1 digestion. Protein was then loaded onto a Superdex 200 gel filtration column for size separation. The protein composition of each fraction was determined by SDS-PAGE analysis. Fractions containing dTH were collected, and EDTA was added to a final concentration of 1 mM to remove divalent metal ions. After incubation at 4 °C for 30 min, the purified protein was concentrated by Amicon® Ultra Centrifugal Filters (type 30,000 NMWL). Then, the protein was diluted 10-fold with EDTA-free 50 mM MES buffer (pH 6.5) and applied to Ultra filters again to be concentrated to its original volume. The procedure was repeated for five times to remove EDTA in the protein buffer.

Competitive binding assays were performed as described previously with some modification [52]. Briefly, purified dTH (2.5 μM subunit) was incubated with 15 μM FeCl_2_, 0-25 μM ZnCl_2_, and 0.5 mg/mL catalase in 50 mM MES Potassium (pH 6.5) for 20 min at 4 °C. After incubation, the mixture was applied to Amicon Ultra filter devices (Merck Millipore). Free and bound metal ions were separated by centrifugation (5 min at 5000 rpm) at 4 °C. The filtrate was collected and contents of iron ion or zinc ion were determined by BPS-based colorimetric assays or ICP-MS. All the experiments were repeated at least three times.

### Determination of L-dopa

For L-dopa assay in *S. cerevisiae*, Catsup and dTH were cloned into pYES2 (Invitrogen) and pYES3 (Invitrogen), respectively. The plasmids were transformed into ZHY3 or wild-type yeast INVSc1. The vectors pYES3 and pYES2 were also transformed into yeast and used as the controls. For L-dopa produced in yeast, a single colony was picked and grown in SD medium at 30 °C to OD_600_ = 1. Then, the yeast cells were collected by centrifugation at 3000*g* for 5 min, washed with sterile water, and transferred to SGR medium supplemented with 0.4 mg/mL tyrosine, 2.0 mg/mL ascorbic acid, and 1 mM DMPH_4_. In the experiments for testing metal effects, 400 mM BPS along with FeSO_4_ or ZnCl_2_ was added in the medium. The yeast was shaken at 30 °C for 18 h, and the supernatant was collected for L-dopa level determination.

For L-dopa assays in *E.coli*, pET28a-His6-SUMO-dTH mentioned above was transformed into BL21 (DE3). The vector pET28a was transformed into BL21(DE3) as control. Protein expression was induced by 250 μM IPTG at 37 °C. The LB medium was supplied with 400 μg/mL tyrosine, 2.0 mg/mL ascorbic acid, and 1 mM DMPH_4_ for L-dopa production. FeSO_4_ or ZnCl_2_ was added as indicated in each experiment. L-dopa level was determined after 8 h growth at 37 °C. L-dopa level was determined as previously described [72] with some modifications. Briefly, the colorimetric method for estimation of L-DOPA was performed in 96-well microtiter plates. Fifty microliters each of 0.5 M HCl, nitrite-molybdate reagent (composed of 10% w/v sodium nitrite and 10% w/v sodium molybdate), and 1.0 M NaOH was sequentially added to 50 μL of the sample. The absorbance was measured at 530 nm using a Thermo Multiskan GO reader. A calibration curve with different amounts of L-dopa was constructed. L-dopa level in each sample was calculated based on the standard curve or normalized to the control. All experiments were repeated at least three times.

### Dopamine level determination

For sample preparation, 3rd larvae were collected and sonicated in 0.6 mL of ice-cold 0.4 M perchloric acid. After centrifugation at 13000*g* at 4 °C for 10 min, the supernatant was transferred into a new tube and mixed with a half supernatant volume of 20 mM potassium citrate, 300 mM K_2_HPO_4_, and 2 mM EDTA. The mixture was kept on ice in the dark for 20 min. Then, the chilled mixture was filtered through a low-binding Durapore (0.22 μm) PVDF membrane. The amounts of dopamine were measured by an HPLC-ECD system. Chromatographic separation was performed on a Diamonsil C18 column (5 μm, 250 × 4.6 mm). The mobile phase contained 60 mM KH_2_PO_4_, 350 μM C_8_H_19_NaO_4_S, 100 μM EDTA, and 8% formaldehyde. Dopamine levels were monitored using a HPLC system (Water e2695) equipped with an electrochemical detector (Water 2645). The experiments were repeated three times.

### TH activity assay

TH activity was determined as previously described with some modifications [73, 74]. For the TH activity assay in *Drosophila* heads, samples were collected and homogenized in 20 mM Tris-HCl buffer, pH 7.5 by ultrasonic disintegration over ice. Protein concentrations were determined with BCA kit and adjusted for each group. Fifty microliters homogenate was further diluted with 300 μL 30 mM Tris-acetic acid containing 0.1% Triton X-100 and 25 μCi of radioactive tyrosine HCl (0.4 mCi/nMol of L-[ring-3,5-^3^H]tyrosine), 50 nMol of the cofactor 6, 7-dimethyl 5, 6, 7, 8-tetrahydropterin (DMPH_4_, Sigma, St. Louis, MO, USA), 5000 units of catalase, and 5 nM DTT in 100 mM potassium phosphate, pH 6.0 for incubation at 37 °C for 20 min. Then, aliquots were taken and placed in 5% (v/v) perchloric acid to stop the reaction. Unreacted tyrosine and the product DOPA were absorbed with 1.0 mL of an aqueous slurry of activated charcoal (in 0.1 M HCl) and the released [^3^H]H_2_0 was analyzed by liquid scintillation counting. TH utilizes O_2_ to produce DOPA, and [^3^H]H_2_0 is derived from [^3^H]tyrosine. The TH activity was expressed as radiation value of [^3^H]H_2_0 produced per group.

For the TH activity assay in yeast, yeast was cultured in SD media at 30 °C for 24 h to OD *=* 1. Yeast was collected by centrifugation at 3000*g* for 5 min and then transferred to SGR media supplemented with metals or metal chelators, as indicated in each experiment; 500 nMol DMPH_4_ (Sigma, St. Louis, MO, USA) and 10 μL L-[ring-3,5-^3^H]tyrosine were added in the media and then the yeast was further cultured at 30 °C for 24 h. Cells were removed after centrifugation at 12,000*g* for 15 min. The supernatant was transferred to a new container. Unreacted tyrosine and the product DOPA were absorbed with 1.0 mL of an aqueous slurry of activated charcoal (in 0.1 M HCl) and the released [^3^H]H_2_0 was analyzed by liquid scintillation counting. The TH activity was expressed as radiation value of [^3^H]H_2_0 produced per group. All experiments were repeated at least three times.

### Statistical analysis

All data were recorded and analyzed with Microsoft Excel and GraphPad Prism (version 6.00; La Jolla, CA, USA). Data were analyzed by Student’s *t-*test between the groups, and ANOVA was used for multiple comparison. Statistical results were presented as means ± SEM. Asterisks indicate critical levels of significance (*p < 0.05, **p < 0.01, ***p < 0.001).

## Supplementary Information

**Additional file 1: Figure S1.** Quantification of the signal intensity in Figure 4B. No obvious and consistent changes were observed. All values are presented as mean ± SEM and included in Additional file 2: table S13; *n*=4. **Figure S2.** L-dopa production is promoted by iron and suppressed by zinc in *INVSc1*. (A) The generation of L-dopa in *INVSc1* was elevated by iron and inhibited by zinc added in the iron deficient medium. All values are presented as mean ± SEM and included in Additional file 2: table S14; n=3. *p<0.05, **p<0.01,***p<0.001. (B) L-dopa production in *INVSc1* was inhibited by Catsup expression in the iron abundant medium. All values are presented as mean ± SEM and included in Additional file 2: table S15; n=3. *p<0.05. **Figure S3.** TH protein levels were not much changed under metal treatments. A coomassie blue staining of protein gel was performed to test the effect of iron and zinc on the expression of TH in *E. coli*. **Figure S4.** ICP-MS indicated that zinc competes with iron in binding with *Drosophila* TH. Zinc binding sites could exceed 1 at high zinc concentrations, possibly due to additional non-specific bindings. All values presented in this graph are included in Additional file 2: table S12. **Figure S5.** Effects of modulating zinc and iron homeostasis on the mobility and survival of flies in the absence of rotenone. (A) The mobility of flies of various genotypes raised on normal food. n=6. ***p<0.001. (B) The lifespan of flies of various genotypes in the absence of rotenone. Genotypes in (A) and (B) of the flies used were *TH-Gal4>w*^*1118*^ (control), *TH-Gal4>Catsup RNAi*, *TH-Gal4>Catsup OE*, *TH-Gal4>dZnT7 OE*, *TH-Gal4>Mvl OE*. All values are presented as mean ± SEM; n= 6. **Figure S6.** TH phosphorylation in SH-SY5Y cells under different metal or metal chelator treatments. Gel blot was cut around the potential target region as indicated by the markers and then hybridized to TH and P-TH respectively. These are the original gel exposure pictures. Related to Figure 4B.

**Additional file 2.** The individual raw data values of figures and Additional files for number of replicates ≤ 6. All the data are cited in the figure legend.

## Data Availability

All data generated or analyzed during this study are included in this published article and its supplementary information files.

## Abbreviations

DA: Dopamine
PD: Parkinson’s disease
TH: Tyrosine hydroxylase
Catsup: *Catecholamines up*
CNS: Central nervous system
OE: Overexpression
ALP: Alkaline phosphatase
TPEN: *N*,*N*,*N*′,*N*′-tetrakis(2-pyridylmethyl)ethylenediamine
BPS: Bathophenanthrolinedisulfonic acid
BH4: Tetrahydrobiopterin
DMPH4: 6, 7-Dimethyl 5, 6, 7, 8-tetrahydropterin
GTPCH: GTP cyclohydrolase I
ICP-MS: Inductively coupled plasma mass spectrometry
