# Supplementary material for: Zinc antagonizes iron-regulation of tyrosine hydroxylase activity and dopamine production in Drosophila melanogaster
Source: BMC Biol. 2021 Nov 3;19:236. doi: 10.1186/s12915-021-01168-0 (PMC8564973; doi:10.1186/s12915-021-01168-0)
Supplement: Supplementary file 1 — Additional file 1: Figure S1. Quantification of the signal intensity in Figure 4B. No obvious and consistent changes were observed. All values are presented as mean ± SEM and included in Additional file 2: table S13; n=4. Figure S2. L-dopa production is promoted by iron and suppressed by zinc in INVSc1. (A) The generation of L-dopa in INVSc1 was elevated by iron and inhibited by zinc added in the iron deficient medium. All values are presented as mean ± SEM and included in Additional file 2: table S14; n=3. *p<0.05, **p<0.01,***p<0.001. (B) L-dopa production in INVSc1 was inhibited by Catsup expression in the iron abundant medium. All values are presented as mean ± SEM and included in Additional file 2: table S15; n=3. *p<0.05. Figure S3. TH protein levels were not much changed under metal treatments. A coomassie blue staining of protein gel was performed to test the effect of iron and zinc on the expression of TH in E. coli. Figure S4. ICP-MS indicated that zinc competes with iron in binding with Drosophila TH. Zinc binding sites could exceed 1 at high zinc concentrations, possibly due to additional non-specific bindings. All values presented in this graph are included in Additional file 2: table S12. Figure S5. Effects of modulating zinc and iron homeostasis on the mobility and survival of flies in the absence of rotenone. (A) The mobility of flies of various genotypes raised on normal food. n=6. ***p<0.001. (B) The lifespan of flies of various genotypes in the absence of rotenone. Genotypes in (A) and (B) of the flies used were TH-Gal4>w1118 (control), TH-Gal4>Catsup RNAi, TH-Gal4>Catsup OE, TH-Gal4>dZnT7 OE, TH-Gal4>Mvl OE. All values are presented as mean ± SEM; n= 6. Figure S6. TH phosphorylation in SH-SY5Y cells under different metal or metal chelator treatments. Gel blot was cut around the potential target region as indicated by the markers and then hybridized to TH and P-TH respectively. These are the original gel exposure pictures. Related to Figure 4B. [file 12915_2021_1168_MOESM1_ESM.docx]

**Additional file 1**


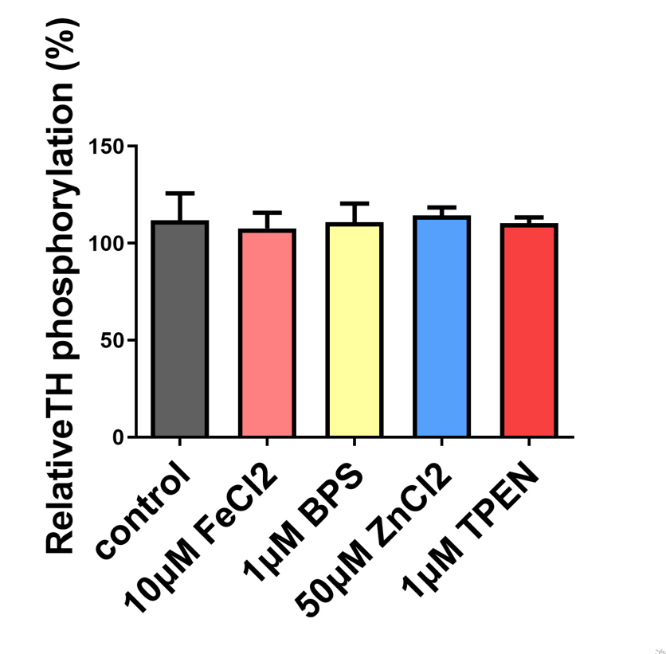


**Figure S1 Quantification of the signal intensity in Figure 4B.** No obvious and consistent changes were observed; All values are presented as mean ± SEM and included in Additional file 2: table S13; n=4.


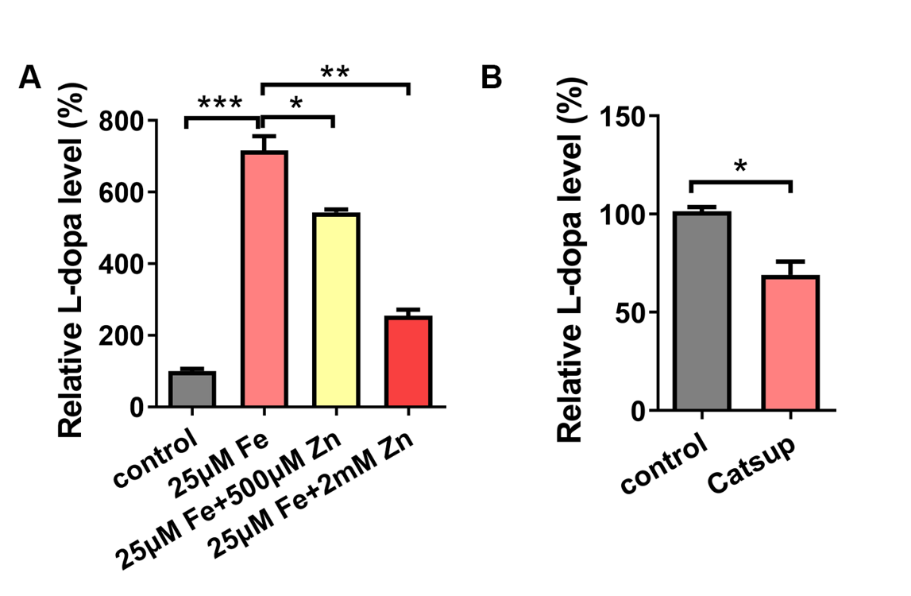


**Figure S2 L-dopa production is promoted by iron and suppressed by zinc in *INVSc1*.**

(A) The generation of L-dopa in *INVSc1* was elevated by iron and inhibited by zinc added in the iron deficient medium. All values are presented as mean ± SEM and included in Additional file 2:table S14; n=3. *p<0.05, **p<0.01,***p<0.001.

(B) L-dopa production in *INVSc1* was inhibited by Catsup expression in the iron abundant medium. All values are presented as mean ± SEM and included in Additional file 2:table S15; n=3. *p<0.05.


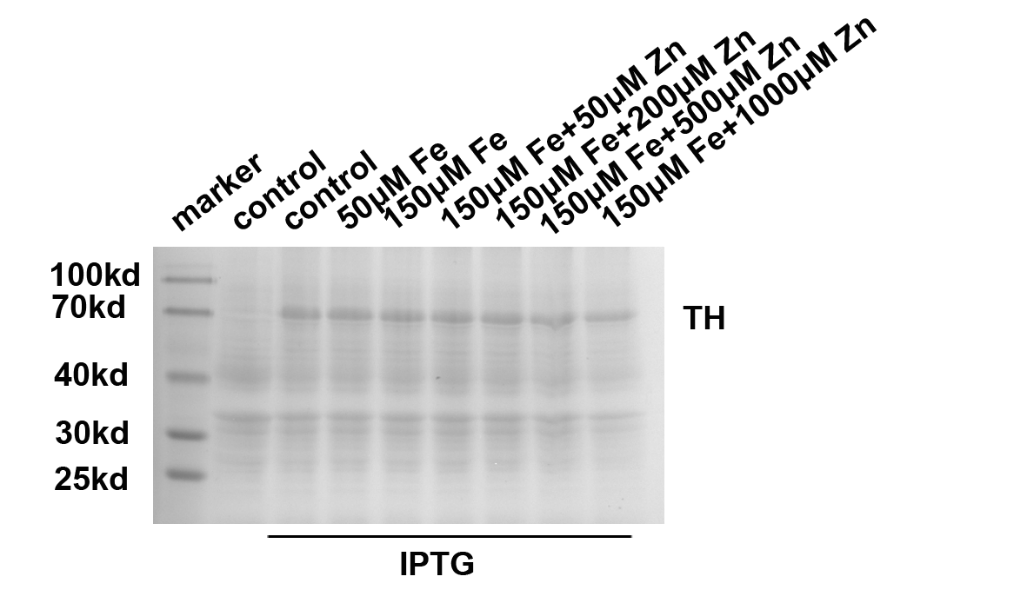


**Figure S3 TH protein levels were not much changed under metal treatments**. A coomassie blue staining of protein gel was performed to test the effect of iron and zinc on the expression of TH in *E. coli*.


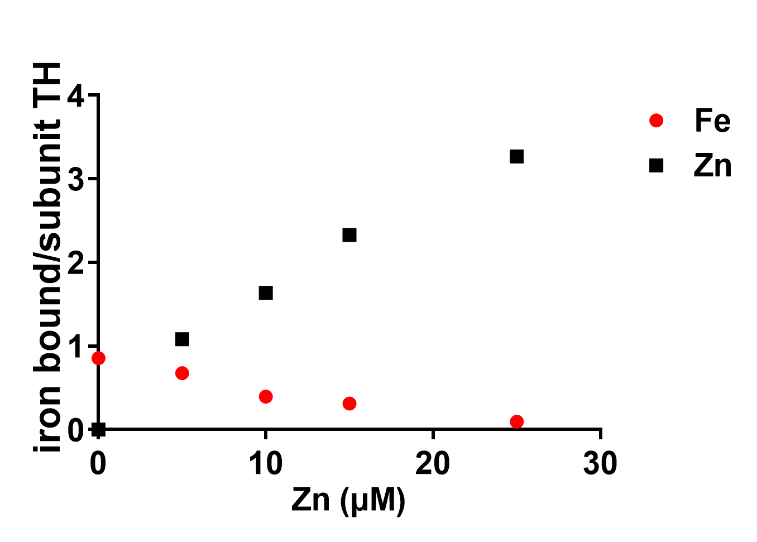


**Figure S4 ICP-MS indicated that zinc competed with iron in binding with Drosophila TH.** Zinc binding sites could exceed 1 at high zinc concentrations, possibly due to additional non-specific binding. All values presented in this graph are included in Additional file 2: table S12.


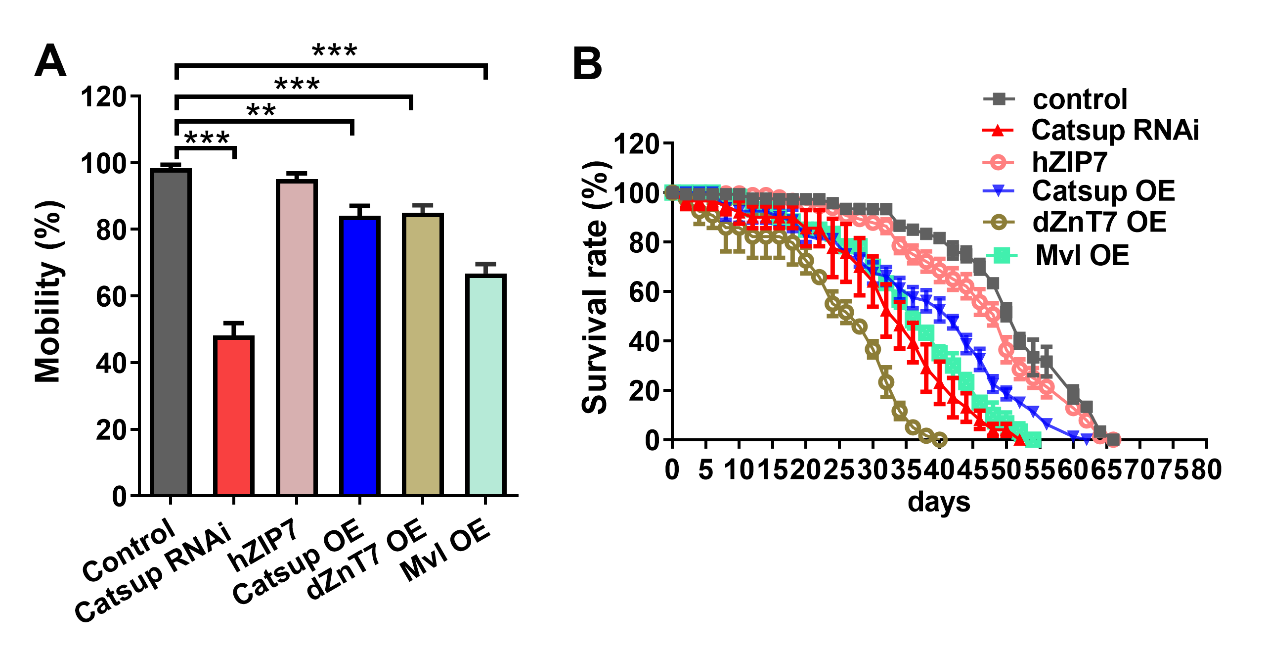


**Figure S5 Effects of modulating zinc and iron homeostasis on the mobility and survival of flies in the absence of rotenone.**

(A) The mobility of flies of various genotypes raised on normal food. n=6. ***p<0.001.

(B) The lifespan of flies of various genotypes in the absence of rotenone. Genotypes in (A) and (B) of the flies used were *TH-Gal4>w^1118^* (control), *TH-Gal4>Catsup RNAi*, *TH-Gal4>Catsup OE*, *TH-Gal4>dZnT7 OE*, *TH-Gal4>Mvl OE*. All values are presented as mean ± SEM; n= 6


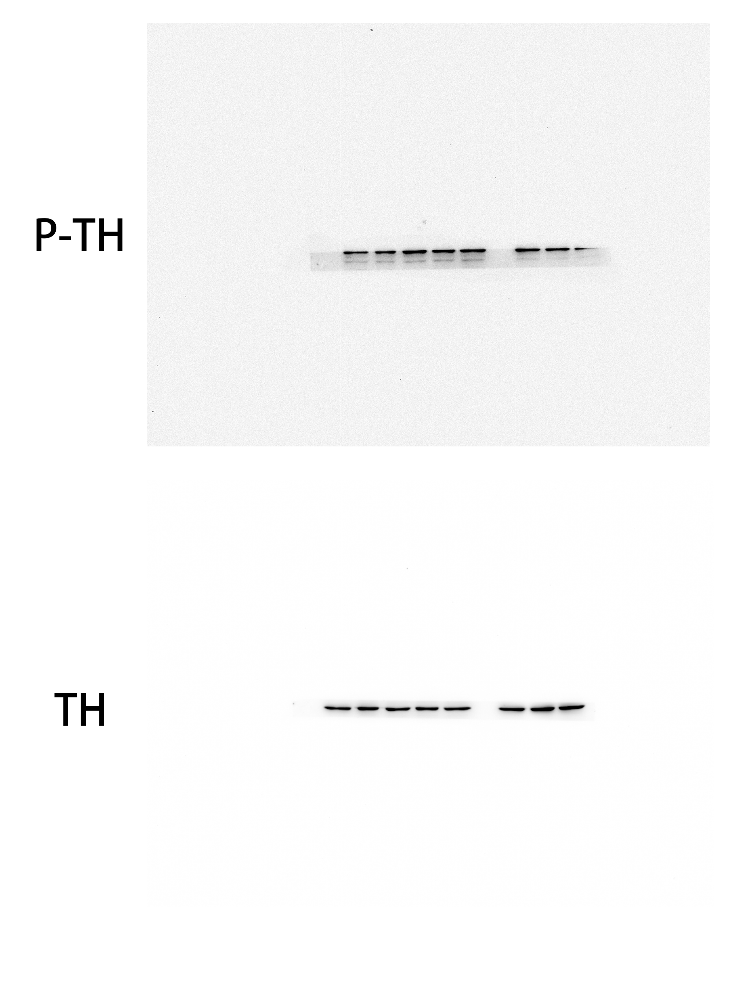


**Figure S6 TH phosphorylation in SH-SY5Y cells under different metal or metal chelator treatments.** Gel blot was cut around the potential target region as indicated by the markers and then hybridized to TH and P-TH respectively. These are the original gel exposure pictures. **Related to figure 4B.**
